# Predictive Machine Learning Models and Survival Analysis for COVID-19 Prognosis Based on Hematochemical Parameters

**DOI:** 10.3390/s21248503

**Published:** 2021-12-20

**Authors:** Nicola Altini, Antonio Brunetti, Stefano Mazzoleni, Fabrizio Moncelli, Ilenia Zagaria, Berardino Prencipe, Erika Lorusso, Enrico Buonamico, Giovanna Elisiana Carpagnano, Davide Fiore Bavaro, Mariacristina Poliseno, Annalisa Saracino, Annalisa Schirinzi, Riccardo Laterza, Francesca Di Serio, Alessia D’Introno, Francesco Pesce, Vitoantonio Bevilacqua

**Affiliations:** 1 Department of Electrical and Information Engineering (DEI), Polytechnic University of Bari, 70126 Bari, Italy; nicola.altini@poliba.it (N.A.); antonio.brunetti@poliba.it (A.B.); stefano.mazzoleni@poliba.it (S.M.); f.moncelli1@studenti.poliba.it (F.M.); i.zagaria@studenti.poliba.it (I.Z.); berardino.prencipe@poliba.it (B.P.); e.lorusso3@studenti.poliba.it (E.L.); 2 Apulian Bioengineering SRL, Via delle Violette, 14, 70026 Modugno, Italy; 3 Institute of Respiratory Disease, Department of Basic Medical Science, Neuroscience, and Sense Organs, University of Bari Aldo Moro, 70124 Bari, Italy; enricobuonamico@gmail.com (E.B.); elisa.carpagnano@uniba.it (G.E.C.); 4 Clinic of Infectious Diseases, Department of Biomedical Sciences and Human Oncology, University of Bari Aldo Moro, 70124 Bari, Italy; davidebavaro@gmail.com (D.F.B.); polisenomc@gmail.com (M.P.); annalisa.saracino@uniba.it (A.S.); 5 Clinic Pathology Unit, University Hospital of Bari, 70124 Bari, Italy; annalisa.schirinzi@policlinico.ba.it (A.S.); riccardo.laterza@uniba.it (R.L.); francesca.diserio@policlinico.ba.it (F.D.S.); 6 Internal Medicine Unit, Ostuni Hospital, 72017 Ostuni, Italy; ale_dintrono@libero.it; 7 Nephrology Unit, Department of Emergency and Organ Transplantation (DETO), University of Bari Aldo Moro, 70124 Bari, Italy; francesco.pesce@uniba.it

**Keywords:** COVID-19, machine learning, Kaplan–Meier, Cox regression, hematochemical parameters, prognostic markers

## Abstract

The coronavirus disease 2019 (COVID-19) pandemic has affected hundreds of millions of individuals and caused millions of deaths worldwide. Predicting the clinical course of the disease is of pivotal importance to manage patients. Several studies have found hematochemical alterations in COVID-19 patients, such as inflammatory markers. We retrospectively analyzed the anamnestic data and laboratory parameters of 303 patients diagnosed with COVID-19 who were admitted to the Polyclinic Hospital of Bari during the first phase of the COVID-19 global pandemic. After the pre-processing phase, we performed a survival analysis with Kaplan–Meier curves and Cox Regression, with the aim to discover the most unfavorable predictors. The target outcomes were mortality or admission to the intensive care unit (ICU). Different machine learning models were also compared to realize a robust classifier relying on a low number of strongly significant factors to estimate the risk of death or admission to ICU. From the survival analysis, it emerged that the most significant laboratory parameters for both outcomes was *C-reactive protein min*; HR=17.963 (95% CI 6.548–49.277, *p* < 0.001) for death, HR=1.789 (95% CI 1.000–3.200, *p* = 0.050) for admission to ICU. The second most important parameter was *Erythrocytes max*; HR=1.765 (95% CI 1.141–2.729, *p* < 0.05) for death, HR=1.481 (95% CI 0.895–2.452, *p* = 0.127) for admission to ICU. The best model for predicting the risk of death was the decision tree, which resulted in ROC-AUC of 89.66%, whereas the best model for predicting the admission to ICU was support vector machine, which had ROC-AUC of 95.07%. The hematochemical predictors identified in this study can be utilized as a strong prognostic signature to characterize the severity of the disease in COVID-19 patients.

## 1. Introduction

In December 2019, in Wuhan, province of Hubei (China), several local health facilities reported cases of pneumonia of unknown origin, which have been identified as the first human cases of COVID-19 [1,2]. The SARS-CoV-2 virus pandemic has caused more than 5,000,000 deaths and a total of over 250,000,000 confirmed cases, globally, as of November 2021 [3,4].

Most patients have mild, self-limiting respiratory infections, with symptoms such as fever, headache, dry cough, fatigue, and muscle pain, but some may rapidly develop fatal complications, including acute respiratory distress syndrome (ARDS) or respiratory failure, multiple organ dysfunction, and septic shock that imposes hospitalization and could lead to the death of the patient [1,5].

This pandemic has put a strain on all global health systems and represents a formidable opportunity to highlight the value of laboratory medicine and to focus on new methods to support and speed up the identification of patients with higher risks of progression to severe stages of the disease.

Accurate prediction of COVID-19 mortality and the identification of factors related to the severity of the disease would allow for targeted strategies in those patients with higher risk of death or developing severe disease; thus, reducing the burden of unnecessary hospitalizations and the health system overload [6].

A better (and clearer) understanding of predictive factors for COVID-19 is crucial for the development of clinical decision support systems that can accurately and rapidly detect the patients with increased risk of worsening conditions [7].

Towards this aim, we retrospectively analyzed data from a cohort of 303 patients with reverse transcription-polymerase chain reaction (RT-PCR) confirmed COVID-19, hospitalized at Polyclinic Hospital of Bari, during the first phase of the COVID-19 global pandemic from 14 March to 10 September 2020. Statistical methods and survival analysis, together with the development of machine learning classifiers, were carried out on these data, with the purpose of identifying hematochemical parameters that better reflect and contribute to the risk assessment.

The paper is structured as follows. Section 2 summarizes the relevant literature on the predictive models for COVID-19. Section 3 describes the details of the data collection process, the patient cohort, and the analysis framework. Section 4 details the methods exploited for carrying out the analysis, and explains the feature selection process and the development of machine learning (ML) classifiers for the risk assessment, considering both the death and the admission to the intensive care unit (ICU) as target outcomes. As for the admission to the ICU, we included patients who were admitted at the start to the ICU or were transferred to the ICU from the other COVID Units. In Section 5, we present and discuss the obtained results. Lastly, in Section 6, we summarize the findings of this research.

## 2. Related Works

Different authors considered the task of performing statistical analysis or developing ML models to predict the severity of COVID-19 disease [8,9,10,11,12,13,14,15,16,17,18]. Tjendra et al. [12] performed a meta-analysis, which summarize 72 papers on the predictive role of different biomarkers in COVID-19 patients. According to them, white blood cells, lymphocyte and platelet counts, C-reactive protein (CRP), ferritin, and interleukin-6 were found to be potential prognostic markers of evolution of the disease to a severe form.

Yoshida et al. [8] discovered sex disparities in clinical and biological parameters of severe outcomes in 776 adults with COVID-19, hospitalized in a U.S. healthcare system. The data from the cohort were acquired in New Orleans, LA, between 27 February and 15 July 2020.

Nachtigall et al. [9] retrospectively analyzed 1904 patients admitted to a national network of hospitals in Germany. The authors considered demographic data, comorbidities, and clinical outcomes, and revealed that the most important risk factors for death were older age, precedent lung disease, and male sex.

Banoei et al. [10] performed a multivariate predictive analysis on a subset of 108 out of 250 features, encompassing comorbidities, blood markers, and clinical features. The features considered were those captured at the admission time from a cohort of 250 hospitalized patients with COVID-19. The strongest mortality predictors were diabetes, coronary artery disease, altered mental status, dementia and age greater than 65 years. Among the biochemical markers, the most relevant were CRP, lactate, and prothrombin.

Zuccaro et al. [11] considered a cohort of 426 consecutive hospitalized patients from a hospital in Lombardy, Italy, in the period 12 February–30 March 2020. They concluded that male sex, older age, hospital admission after 4 March, and number of comorbidities were independent risk factors related to in-hospital mortality.

Zhou et al. [13] retrospectively analyzed 116 patients admitted to Chongqing Public Health Medical Center, China, in the period 24 January–7 February, 2020, with a diagnosis of mild or moderate COVID-19. According to the authors, three factors were found to be independent predictors of progression to severe disease, during two weeks after admission: high value of creatine kinase, low value of CD4+ T-cell count, and age higher than 65 years.

Niu et al. [14] included a cohort of 150 patients diagnosed with COVID-19 from Huanggang Central Hospital in the period 23 January–5 March, 2020. By exploiting univariate and multivariate logistic regression, the authors explored which were the most relevant risk factors associated with in-hospital death. This analysis allowed concluding that diabetes, high value of lactate dehydrogenase on admission, and higher sequential organ failure assessment score increased the odds of in-hospital death. A summary of the related works is available in Table 1.

Deep learning (DL) approaches are becoming more relevant in the biomedical and health domains, and literature already exists for what concerns the COVID-19 pandemic [19]. Even though most of the literature focuses on tasks, such as medical image analysis, biomedical signal processing, and natural language processing, which are domains different from ours, there is a recent trend in exploiting DL models for irregularly sampled time series (ISTS) data. Sun et al. performed a review of the DL methods for addressing the issues arising from ISTS data [20]. They also consider a COVID-19 dataset, coming from the work of Yan et al. [21], for which they discover that, for mortality prediction, T-LSTM [22] and GRU-D [23] are the top performing models. With respect to DL approaches, the statistical and machine learning framework developed in this paper more easily allows one to interpret the results, also from a clinical significance point of view.

Most of the available works in the literature are considered demographic data, comorbidities, and blood markers. In this work, our purpose was to realize a predictive model based on hematochemical parameters. Unlike what was done in previous works, as Banoei et al. [10], which considered blood markers at admission time, we included time series data for hematochemical factors, allowing the construction of a more reliable predictive model. Niu et al. [14] considered the evolution of parameters over time, but based their conclusions on a cohort smaller than ours, being composed of only 150 patients. As predictive models, they mainly considered univariate and multivariate logistic regression, whereas we compared a wide variety of methods: Decision tree (DT), random forest (RF), Gaussian naive Bayes (GNB), support vector machines (SVM), K-nearest neighbors (KNN), and adaptive boosting. Finally, other authors, as Nachtigall et al. [9], did not consider blood parameters in their analyses. Therefore, our paper can be considered a contribution over the existing literature, especially because we performed, in a cohort of 303 patients, statistical and survival analyses and systematic comparison of predictive models over time series of hematochemical parameters.

## 3. Materials

### 3.1. Data Collection

The demographic and anamnestic data were collected by clinicians and specialists from four different COVID-Units of the Polyclinic Hospital of Bari (Apulia, Southern Italy): Intensive Care Unit (41 patients), Infectious Disease Unit (224 patients), Pneumology Unit (122 patients), and Internal Medicine Unit (324 patients). In total, data of 434 patients were collected. Laboratory tests were performed by specialists from the Clinic Pathology Unit of the aforementioned Hospital, providing data of 367 patients. The intersection among demographic, clinical, and laboratory data resulted in a dataset of 303 patients.

Specifically, demographic data included variables, such as age and sex, the clinical characteristics examined were date of hospitalization, record the date of transfer to ICU, date of discharge from all COVID units including the ICU, date of death, days of hospitalization; as for laboratory tests, a total of 69 hematochemical parameters were analyzed. The full list of hematochemical parameters considered for the study is available in supplementary materials.

The target outcomes were in-hospital death and admission to ICU. Events were considered to have occurred only if they happened within the follow-up period.

**Figure 1:**
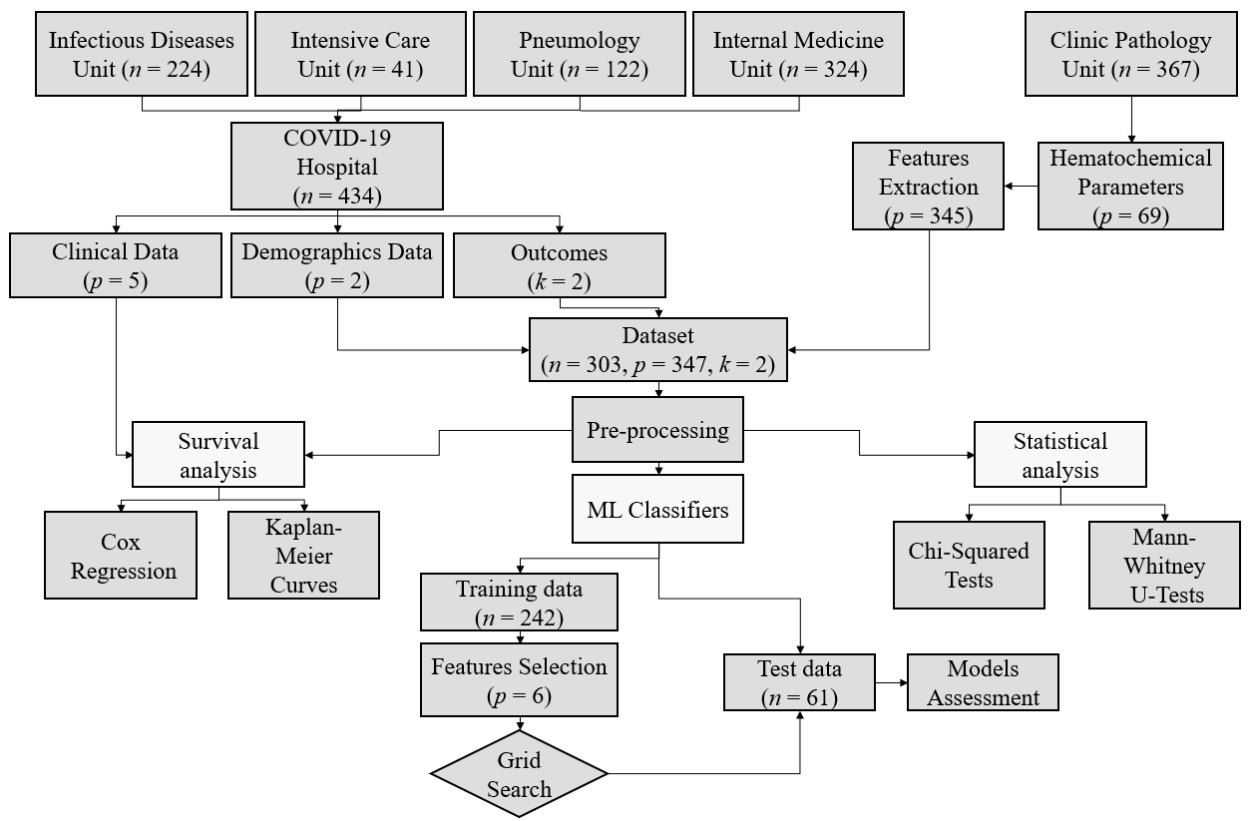
**Data Processing Workflow**. The figure shows the study workflow, starting from the data collection step until the development and assessment of the different predictive models. ML stands for machine learning. Considered ML classifiers include decision trees, random forests, support vector machines, Gaussian naive Bayes, AdaBoost, and K-nearest neighbors.

A workflow of the process followed for carrying out this study, from the data collection to results, is depicted in Figure 1.

### 3.2. Cohort of Study

Overall, 303 patients with COVID-19 were enrolled in the study, of which 184 (60.7%) were male and 119 (39.3%) were female.

The following data are reported as mean ± standard deviation. The age of the study cohort was 64.2 ± 17.7 years (range 19–99 years). The hospitalization time was 22.3 ± 17.1 days (range 0–126 days) and the ICU staying time was 3.7 ± 10.5 days (range 0–94 days).

During the time of hospitalization, 218/303 (71.9%) patients were discharged alive, 85/303 (28.1%) died before discharged, and 74/303 (24.4%) were admitted to the ICU. Among the ICU patients, 49/74 (66.2%) died and 25/74 (33.8%) survived.

On the total of 184 male patients, 54 (29.3%) died, 130 (70.7%) were discharged alive, and 53 (28.8%) were admitted to the ICU, whereas of the 119 female patients, 31 (26.1%) died, 88 (73.9%) were discharged alive, and 21 (17.6%) needed admission to the ICU (Table 2).

The mean age of the dead patients was 74.08 ± 13.15 years, whereas the mean age of the survived patients was 60.36 ± 17.81 years.

In the following, four age classes were considered: under 55 years old, between 55 and 65 years old, between 65 and 80 years old and over 80 years old.

As shown in Table 2, the highest mortality rate was observed in the two oldest age groups (65–80 years and over 80 years), whereas the highest rate of admission or transfer to the ICU was found among patients between 65 and 80 years of age. Patients younger than 55 years and older than 80 years were less likely to be admitted to the ICU.

### 3.3. Analysis Framework

The analysis performed in this study was carried out in the Python 3 programming language. The frameworks exploited included Pandas (for data handling), Scikit-Learn (for training and validating machine learning algorithms), SciPy (to perform the statistical analysis), Seaborn and Matplotlib (to visualize the data).

## 4. Methods

### 4.1. Data Pre-Processing and Data Cleaning

The data collected from the different units were merged into a unique dataset, which we exploited for the following of the study. The obtained dataset contained both (a) demographic and clinical data and (b) hematochemical parameters of the patient cohort. Since, for many laboratory tests examined, there were available time series data, which can allow to understand the time progression of the clinical state, five features were extracted: *minimum*, *maximum*, *mean*, *first*, and *last* values [24].

Outlier removal was performed, considering only the 99.75th percentile values, excluding the remaining 0.25th percentile values, both from upper and lower sides.

For the machine learning predictive models, in order to handle missing values, imputation with the KNNImputer algorithm was performed. It exploits the Euclidean distance to find the nearest neighbors and imputes the missing values with the uniformly averaged values from the specified number of neighbors [25].

Lastly, the data were rescaled into the range [0,1]. This process is useful for features that are not normally distributed and preserves zero entries in sparse data.

According to the literature, the application of these algorithms should lead to an increase of the machine learning classifiers performance [26].

### 4.2. Statistical Analysis

The variables of interest were divided into quantitative variables, i.e., continuous variables that contain numerical values, such as age, and the minimum, average, maximum, first and last values of each hematochemical parameter examined, and qualitative variables, i.e., variables describing the patient’s status as sex, death, or admission to the ICU.

**Descriptive statistics**. Regarding categorical variables, absolute and relative frequencies have been considered. While, regarding continuous variables, mean, median, first quartile, second quartile, third quartile, and interquartile range have been extracted.

**Inferential statistics**. Inferential statistics was carried out using the Chi-squared test for the categorical variables and the Mann–Whitney U test for the continuous variables. For both kind of tests, the significance threshold was set to 0.05. Even though some debate exists about thresholds for *p*-value [27], 0.05 is the historical and the most widely adopted threshold for testing statistical significance. In order to make our work comparable with the majority of existing literature, we decided to adopt the same threshold.

### 4.3. Survival Analysis

Survival analysis corresponds to a set of statistical methodologies used to model and analyze temporal data, in order to investigate the time required for the occurrence of the event under study.

In this study, the Kaplan–Meier method has been exploited for categorical variables (i.e., age classes and sex) to estimate the survival time and generate survival curves, which were obtained by plotting the survival probabilities in relation to the hospitalization days for both outcomes, i.e., in-hospital mortality and admission to ICU [28].

Instead, Cox regression was applied for the blood parameters, considering the laboratory normality ranges. It is a powerful technique to study the impact of several risk factors on patients’ survival at the same time.

In Cox regression, the dependent variable is the incidence rate of a given event considered as the number of events per person in the time between the entry into the study and the date of the last observation [29]. The events under consideration were death and admission to the ICU.

### 4.4. Feature Selection

The feature selection process consists of choosing a subset of relevant features in order to use machine learning methods effectively, speeding up the algorithms, increasing the prediction accuracy and the comprehensibility of the data [30].

For the features selection step, coefficients resulting from a multivariate logistic regression applied to the two different outcomes were exploited [31].

Considering the logistic regression in Equation (1):

(1) $$p=\frac{1}{1+e^{-(\beta_{0}+\beta_{1}x_{1}+\beta_{2}x_{2}+\ldots +\beta_{k}x_{k})}}$$

where *k* is the number of predictors. The features are preserved only if their respective coefficients meet the criteria in Equation (2):

(2) $$|\beta_{i}|>|mean\left(\left[\beta_{1},\ldots ,\beta_{k}\right]\right)|+std\left(\left[\beta_{1},\ldots ,\beta_{k}\right]\right)$$

where $|\beta_{i}|$ is the absolute value of the *i*-th coefficient $\beta_{i}$, $mean(\left[\beta_{1},\ldots ,\beta_{k}\right])$ is the mean of the coefficients and $std(\left[\beta_{1},\ldots ,\beta_{k}\right])$ is the standard deviation of the coefficients. In this way, only the features mostly related to the patient’s outcome have been retained.

### 4.5. Predictive Models and Machine Learning Techniques

**Splitting of the data**. After the pre-processing stage, the dataset resulted in 303 patients and 347 predictors, composed of the five features for each of the 69 hematochemical parameters plus age and sex information. In order to reduce the number of features, a selection has been carried out as described in Section 5.2, resulting in a subset of only six predictors. This dataset has been divided in two subsets, using an 80/20 split, resulting in a training set composed of 242 patients, and a test set composed of 61 patients.

**Predictive models**. In order to analyze the predictive capacity of the selected variables, it was decided to compare different machine learning models. The following six classifiers have been considered:

1. Decision tree [32,33];
2. Random forest [34,35];
3. Gaussian naive Bayes [36];
4. Support vector machines [37];
5. K-nearest neighbors [38];
6. Adaptive boosting or AdaBoost [39,40].

**Models evaluation and settings**. In order to evaluate the models during the hyperparameters exploration, the exhaustive grid search with k-fold cross-validation has been implemented [41]. Final models have been assessed on the hold-out test set. As shown by the literature, this method is used also to improve the classification accuracy [42]. Details about the tuning of hyperparameters with grid search are provided in Appendix A.

The k-fold cross validation has been implemented directly in the grid search and has the advantage of providing a precise estimation of the accuracy of the model and using more data to validate the model [43].

In order to assess the performances of the different models, receiver operating characteristic (ROC) curves and confusion matrices have been exploited.

## 5. Results

### 5.1. Statistical and Survival Analyses

Statistically significant differences in the risk of death, as well as in the risk of admission to the ICU, were found among the age groups, according to the *p*-value < 0.001. Mortality risk was similar for male and female subjects (*p*-value 0.622), whereas statistically significant differences were observed in the risk of admission to the ICU (*p*-value 0.032), with the men more likely to be admitted to the ICU than women. These results are reported in Table 1.

**Figure 2:**
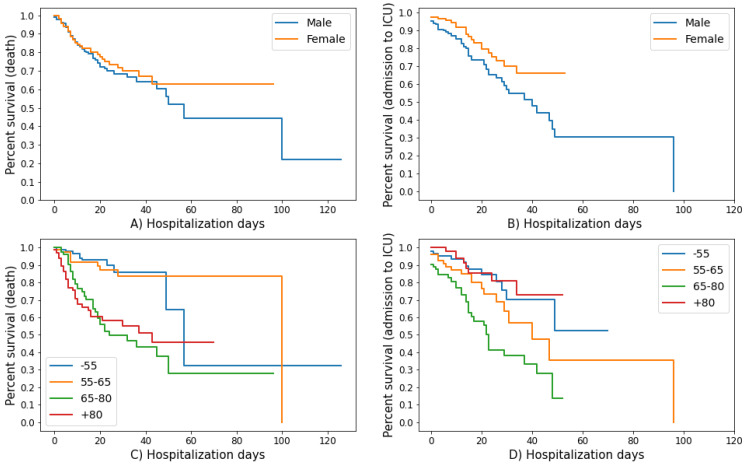
**Kaplan–Meier survival curves**. (**A**) Kaplan–Meier curves for death as a function of hospitalization days stratified by sex. (**B**) Kaplan–Meier curves for the admission to ICU as a function of hospitalization days before the admission stratified by sex. (**C**) Kaplan–Meier curves for death as a function of hospitalization days stratified by age. (**D**) Kaplan–Meier curves for the admission to ICU as a function of hospitalization days before the admission stratified by age.

The Kaplan–Meier survival curves showed a similar survival pattern for males and females (Figure 2A,B). Instead, as shown in Figure 2C,D, divergences in mortality were observed between the younger and the older age groups.

### 5.2. Hematochemical Parameters Analysis

The results of the feature selection process are shown in Table 3 and Table 4, together with the logistic regression coefficients, indicated in the column “**Logit coeff**”. Only features that satisfied Equation (2) have been reported, i.e., features with coefficients higher than the thresholds 2.772 and 3.911, respectively, for mortality and admission to the ICU. From this analysis, 32 features resulted significant for the mortality and 28 features for the admission to the ICU.

In order to extract a unique feature subset, only the features that were found to be significant for both outcomes were retained. They were *Ionized calcium max*, *CRP mean*, *CRP min*, *Total bilirubin min*, *Erythrocyte max*, *Aspartate aminotransferase (AST) min*.

The subset obtained was analyzed using the Mann–Whitney U test to check the statistical significance of each feature; among the six features, three resulted in having a high statistical significance for both outcomes with a *p*-value < 0.05: *CRP mean*, *CRP min*, *Total bilirubin min*.

**Figure 3:**
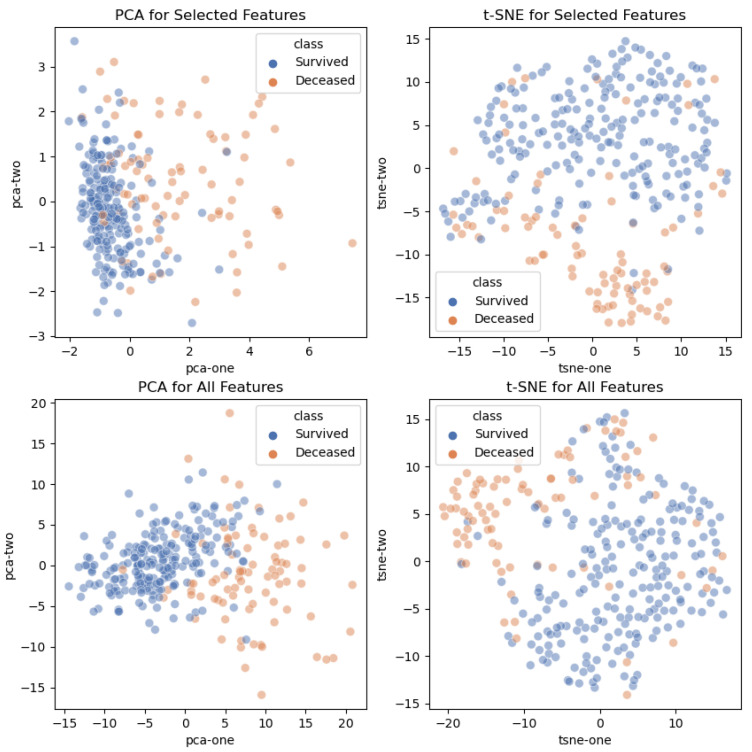
**Scatter plot of low dimensionality feature embedding (death outcome)**. A 2D visualization of hematochemical parameters with PCA and t-SNE. Different colors are used for survived and deceased patients. (Top left) PCA starting from the selected features; (top right) t-SNE from the selected features; (bottom left) PCA starting from all features; (bottom right) t-SNE starting from all features.

**Figure 4:**
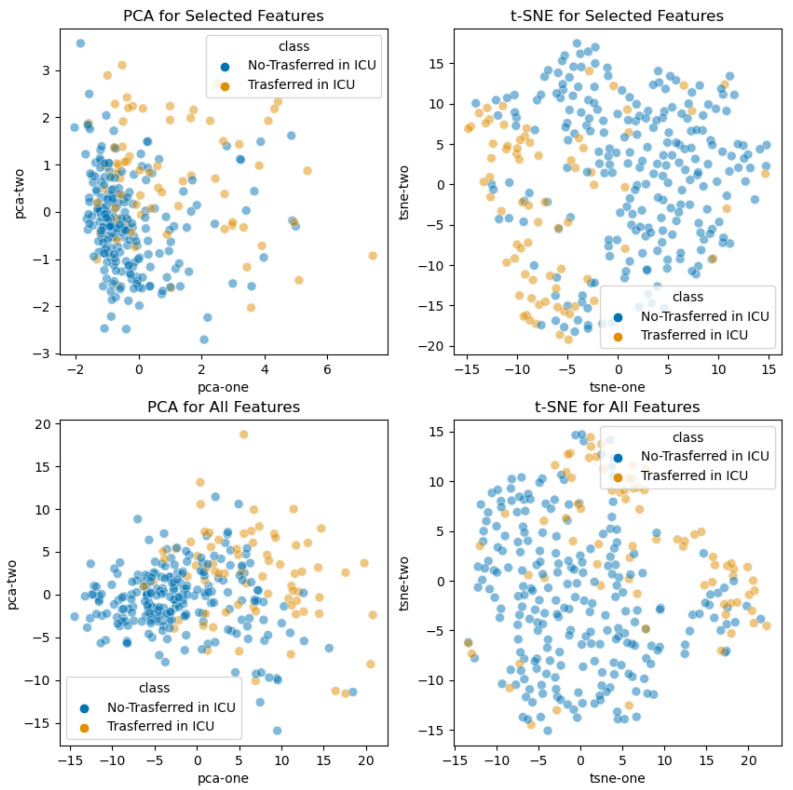
**Scatter plot of low dimensionality features embeddings (admission to ICU outcome)**. A 2D visualization of hematochemical parameters with PCA and t-SNE. Different colors are used for patients, who were (or not) transferred to the ICU. (Top left) PCA starting from the selected features; (top right) t-SNE from the selected features; (bottom left) PCA starting from all features; (bottom right) t-SNE starting from all features.

We also investigated if the considered feature sets, both the starting one with all the features and the other one with the selected prognostic signatures, were discriminative in an embedding scatter plot at reduced dimensionality, exploiting principal component analysis (PCA) and t-distributed stochastic neighbor embedding (t-SNE) [44] techniques. Two plots have been made, one for survived and deceased patients, in Figure 3, and the other one for patients who were or not transferred to the ICU, Figure 4.

**Figure 5:**
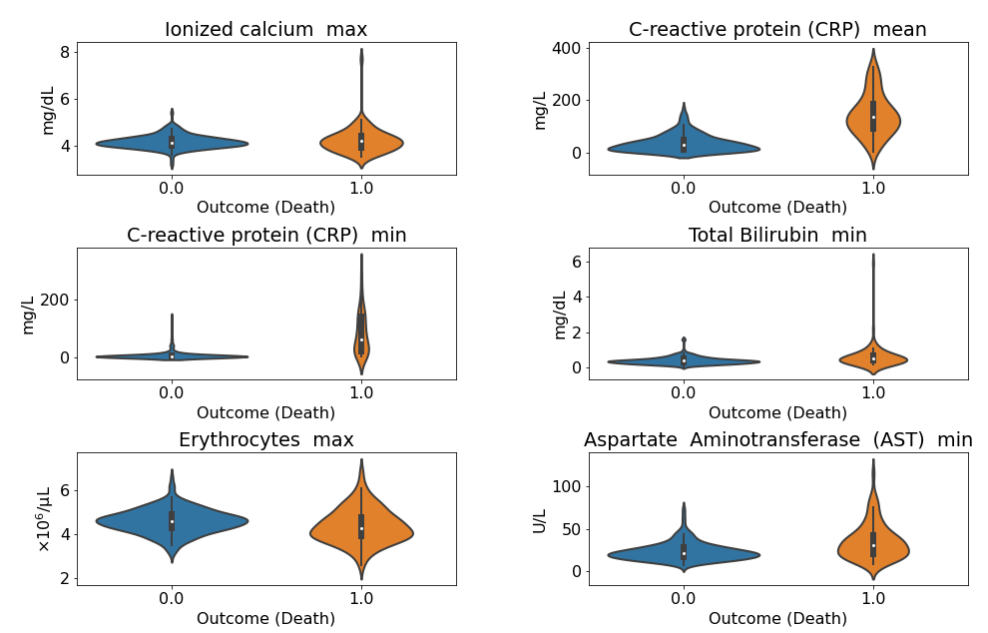
**Violin plots of the distribution of the selected laboratory features considering mortality as outcome**. *C-reactive protein (CRP) mean*, *CRP min*, *Total bilirubin min*, *Erythrocyte max*, *AST min* proved to be statistically significant according to the Mann–Whitney U test.

**Figure 6:**
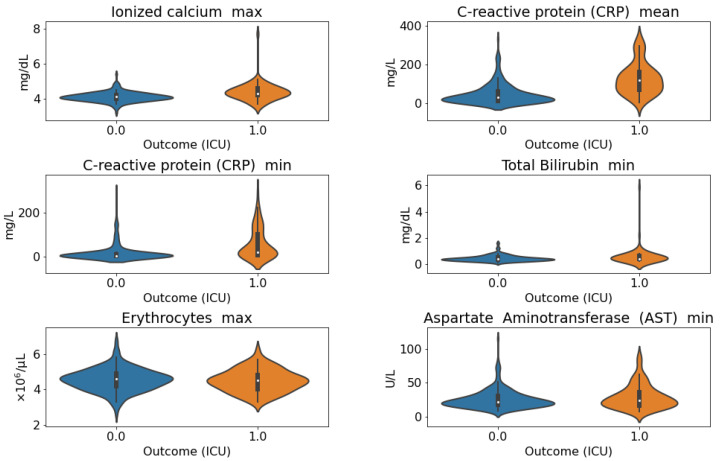
**Violin plots of the distribution of the selected laboratory features considering the admission to ICU as outcome**. *Ionized calcium max*, *CRP mean*, *CRP min*, *Total bilirubin min* proved to be statistically significant, according to the Mann–Whitney U test.

Violin plots that depict the distribution differences between the conditions, both for death and admission to the ICU, are reported in Figure 5 and Figure 6.

Table 5 shows the results of Cox regression analysis used to estimate the relationship between the risk predictive factors, i.e., all the six significant hematochemical features examined, and the mortality rate or the rate of the admission to ICU.

Regarding mortality risk, HR higher than 1 was found for all the six features meaning that patients who had values of the features outside the normality range are at increased risk of mortality. Nonetheless, only the features CRP min and erythrocytes max were statistically significant, with *p* < 0.001 and *p* < 0.05, respectively. It has to be noted that, when we performed the Cox regression analysis, the HR for *CRP mean* was $=3.11\times 10^{6}$ with a 95% CI for log(HR), which spanned from −5020 to 5050, because this feature was overrange in almost every hospitalized patients and 100% of dead patients. In fact the associated *p*-value was 0.995, meaning that its HR was not statistically significant. Therefore, we repeated the Cox regression analysis without this parameter, before reporting the results in Table 5.

Regarding the admission to ICU, HR greater than 1 was observed for all the features, except for *Ionized calcium max*. However, in this case, no feature was statistically significant (*p* < 0.05). The most important predictor was *CRP min*, with HR=1.789 (95% CI 1.000–3.200, *p* = 0.050).

Thus, *CRP min* can be considered the most important risk factor for both outcomes. A medical discussion about these features is provided in Section 5.4.

**Figure 7:**
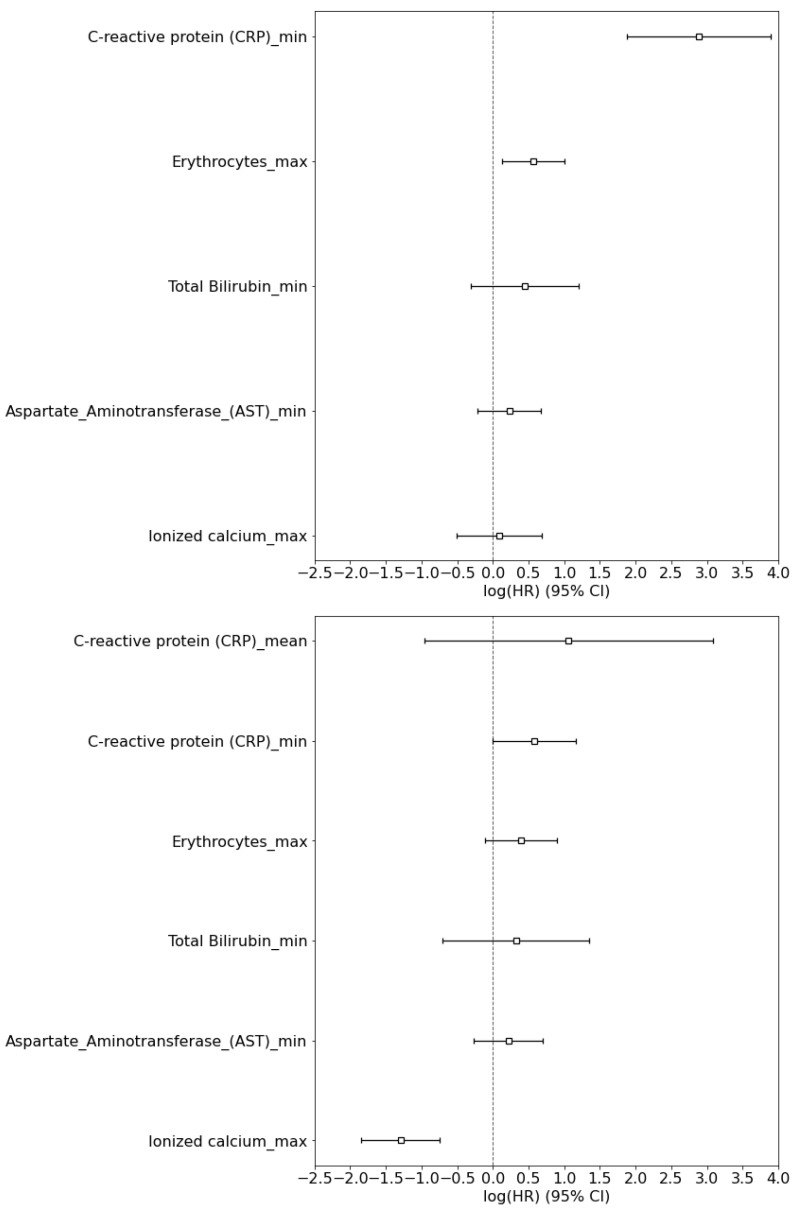
**Cox regression coefficients for mortality risk (top) and risk of admission to ICU (bottom)**. Hazard ratio (HR) is plotted with the 95% confidence interval (CI).

The hazard ratio with the 95% confidence interval for all features is plotted in the logarithmic scale in Figure 7.

### 5.3. Predictive Models

Regarding the predictive models, only the hematochemical parameters have been considered. According to the feature selection stage, only hematochemical tests that resulted significant for both outcomes were retained. They were *Ionized calcium max*, *C-Reactive protein mean*, and *C-reactive protein min*, *erythrocytes max*, *Total bilirubin min* and *aspartate aminotransferase min*.

**Figure 8:**
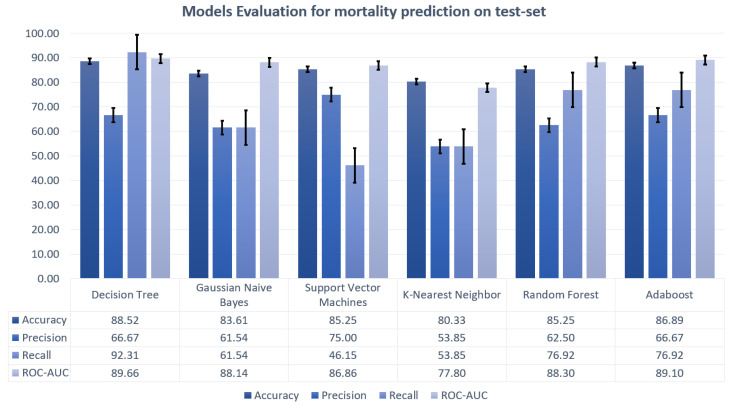
**Predictive model performances for mortality prediction**. Model performances for the mortality prediction displayed as bar plots for accuracy, precision, recall, and ROC-AUC.

**Figure 9:**
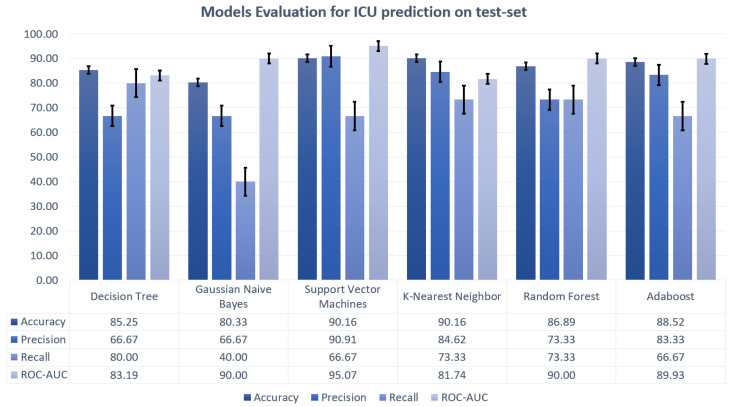
**Predictive model performances for ICU prediction**. Models performances for the ICU admission prediction displayed as bar plots for accuracy, precision, recall, and ROC-AUC.

Machine learning algorithms considered for realizing the predictive models were decision tree, random forest, Gaussian naive Bayes, support vector machines, K-nearest neighbors and AdaBoost, using the exhaustive grid search cross validation to obtain the highest possible accuracy. The performances of the different models are displayed in Figure 8 and Figure 9.

**Figure 10:**
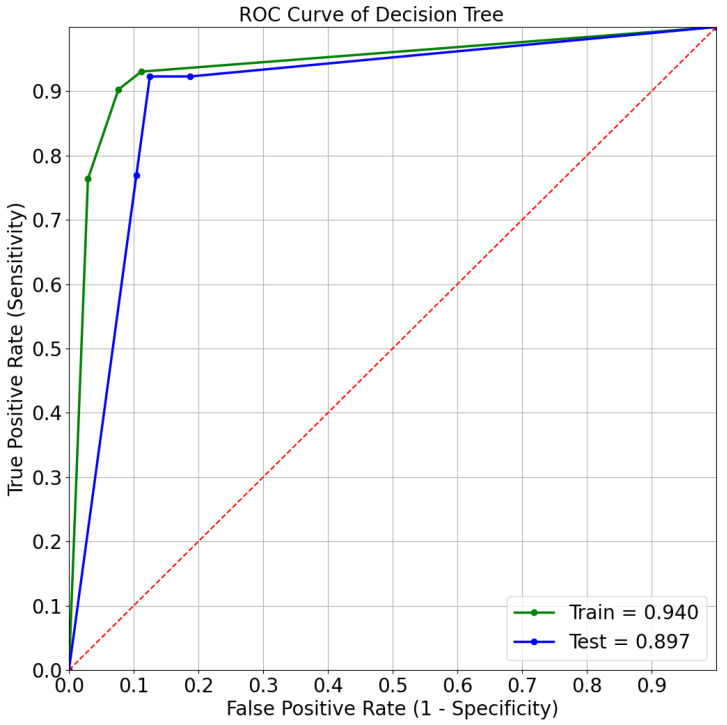
ROC curve of decision tree for mortality prediction.

**Figure 11:**
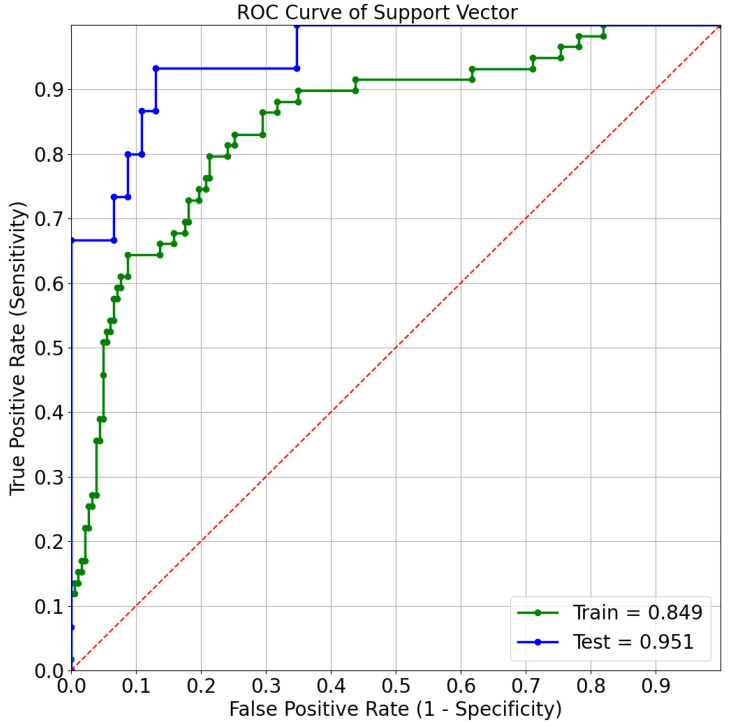
ROC curve of support vector machines for ICU admission prediction.

Decision tree is found to e the model with the highest ROC-AUC for the mortality prediction task, whereas SVM is the best model for predicting admission to ICU. Figure 10 and Figure 11 depict the ROC curves, showing the performances on both the train set and the test set for the best models.

### 5.4. Discussion

These results permit identifying a subset of features that can be used to predict the worsening state of COVID-19.

In the cohort under study, we observed that the patients who were dead or who were admitted to ICU presented alterations of the values of some hematochemical tests that we identify as most predictive factors.

Particularly, we found that the *CRP min* was overrange in 96.4% (41.5%) of the dead (alive) patients and 76.7% (50.4%) of the patients admitted (not admitted) to the ICU, resulting in the main predictor factor for mortality risk and, even not statistically significant, for the risk of admission to the ICU. These data are in accordance with the literature, which suggests that the CRP is strongly associated with mortality in patients with COVID-19 [35,45,46]. On the other hand, it is well known that CRP is a marker for systemic inflammation already associated with severe disease in bacteria or virus infections.

It has been reported that, compared to moderate cases, severe COVID-19 cases had lower red blood cell counts and hemoglobin levels [47]. It has also been stated that COVID-19 is associated to red blood cell (RBC) damage and that the virus negatively affects the process of RBC formation; thus, being responsible for multiple organ damage [48]. Indeed, the statistical analysis showed that, in the cohort of study, the percentage of patients with under range values of erythrocytes max was 45.2% (23.9%) in deceased (alive) patients and 41.1% (26.2%) in patients admitted (not admitted) to the ICU [49]. However, the feature was shown to be only statistically significant for mortality risk.

In our cohort, we also observed that dead patients and patients admitted to the ICU had higher *Total bilirubin min* value compared, respectively, to the survived and patients not admitted to the ICU. Thus, the hyper-bilirubin level can also be exploited as a predictor of worsening conditions in COVID-19 patients. Accordingly, a pooled analysis reported that patients with severe COVID-19 display higher bilirubin levels compared to those with milder forms [50]. An elevated bilirubin level is regarded as a vital marker of altered liver function, indicating a likely liver injury due to the infection [51]. However, hyper-bilirubin levels may be also due to erythrocyte damage and an increased hemolysis rate.

As to the *AST min* value, it was found to be statistically significantly higher in deceased subjects compared to those who were discharged alive. In fact, the extracted min feature was over range, respectively, in 35.7% of dead and 8.8% of survived patients. Likewise the hyper-bilirubin levels, increased AST values, may indicate liver injury due to the SARS-CoV-2 infection and a poorer outcome [52,53].

Finally, the last feature extracted was *Ionized calcium max*, which we found to be under range in a high percentage of patients with COVID-19, irrespective of the severity of the disease. No significant differences were in fact observed between dead and surviving patients. A retrospective case-control study by Pal et al. analyzing 72 patients with non-severe COVID-19 and an equal number of healthy controls reported that hypocalcemia was highly prevalent, even in COVID-19 patients with non-severe disease. They suggest that hypocalcemia may be intrinsic to the disease per se [54]. Cappellini et al. also found a decrease in whole blood ionized calcium levels in COVID-19 versus non-COVID 19 subjects, with the difference being statistically significant [55]. Thus, the lower serum calcium levels observed may be due to a viral direct action on the regulation of the normal ion homeostasis, as shown by the other viruses.

The limitations of the present study are mainly: (a) the acquired cohort comes from a single hospital; therefore, the generalization capability of the developed models—as well as on other cohorts—need to be assessed; (b) only features extracted by time series data of the blood parameters were considered, not the raw data.

## 6. Conclusions and Future Works

Artificial Intelligence can play a pivotal role in processing and analyzing patient data for efficient diagnosis and prognosis. In this paper, we retrospectively analyzed a cohort of hospitalized patients with confirmed diagnoses of COVID, with the purpose of recognizing and evaluating a set of hematochemical parameters, which can be strong predictors of the disease severity, considering, as outcomes, the mortality rate and the rate of admission to ICU.

Starting from the data collection of 303 patients and 347 extracted features, considering five features per each of the 69 hematochemical parameters, in addition to age and sex information, through statistical feature selection techniques, the subset of predictors was reduced to only six features for both target outcomes. They were the *Ionized calcium max*, *CRP mean*, *CRP min*, *Total bilirubin min*, *Erythrocyte max*, *AST min*. We showed that modifications in the value of the six selected predictors are often present in the most severe cases of the disease that are at high risk of deterioration [35,45,46,52,53,55,56,57,58,59,60], with *CRP min* being the main predictor factor.

The best predictive model was the decision tree for the mortality prediction task, with ROC-AUC of 89.66%, and the SVM for the ICU admission prediction, with ROC-AUC of 95.07% confirming the possibility of utilizing these models for both outcome predictions.

In conclusion, the developed models can aid in the realization of a clinical decision support system, which can assist clinicians in the assessment of COVID-19 severity, increasing the precision, accuracy, and velocity of the prediction.

Due to the reliability and accuracy of the developed models, it will be possible to carry out a better stratification risk for COVID-19 hospitalized patients, allowing to reduce severe cases of the disease and deaths.

Future works include the validation of these models on further groups of patients that can allow to better understand the value of the identified predictors. Furthermore, DL models, such as recurrent neural networks (RNNs) [61] or long short-term memory (LSTM) [62], which are architectures designed for modeling temporal sequences, can be exploited to obtain higher accuracy, although at the cost of results that are more difficult to interpret [63].

## Figures and Tables

**Table 1:** Summary of materials and methods exploited in related works.

Authors	Materials			Methods		
	Sample Size	Location	Period	Predictors	Outcomes	Techniques
Yoshida et al.	776 patients	New Orleans, LA	27 February– 15 July 2020	Demographics, comorbidities, presenting symptoms, laboratory results	ICU admission, invasive mechanical ventilation, in-hospital death	Chi-square test, Fischer’s exact test, two tailed *t* test; univariate and multivariate logistic regression.
Nachtigall et al.	1904 patients	Network of Germany Hospitals	12 February– 12 June 2020	Demographics, comorbidities	ICU admission, invasive mechanical ventilation, in-hospital death	Descriptive statistics; survival analysis, multivariate proportional hazard models.
Banoei et al.	250 patients	Miami, FL, USA	since June 2020	Clinical features, comorbidities, blood markers	In-hospital death	SIMPLS (statistically inspired modification of partial least square), PCA, Clustering, Latent class analysis (LCA)
Zuccaro et al.	426 patients	Lombardy, Italy	21 February– 30 March 2020	Demographics, comorbidities, blood markers, treatment, time of hospital admission	In-hospital death, discharge	Student *t* test, Mann–Whitney U test, Chi-square test, DeLong method; Fine and Gray model
Zhou et al.	116 patients	Chongqing, China	24 January– 7 February 2020	Demographics, epidemiological information, clinical manifestation, laboratory test results	Disease progression from milder to severe COVID-19	Chi-square test, Fischer’s exact test, Mann–Whitney U test; Kaplan- Meier; Cox regression.
Niu et al.	150 patients	Huanggang, China	23 January– 5 March 2020	Epidemiological and demographic characteristics, underlying diseases, clinical manifestations, laboratory findings, chest computed tomography (CT) imaging	In-hospital death	Chi-square test, Fischer’s exact test, Mann–Whitney U test; multivariate logistic analysis; nomogram.

**Table 2:** **Demographic characteristics of the patient cohort.** The table displays the demographic characteristics presented as absolute frequency (percentage frequency) of all the patients enrolled in the study.

	Total	Deceased	Survived	Admitted to the ICU	*p*-Value (Mortality)	*p*-Value (ICU)
**Patients**	303	85 (28.1)	218 (71.9)	74 (24.4)		
						
**Sex**					0.6220	0.0384
Male	184 (60.7)	54 (29.3)	130 (70.7)	53 (28.8)		
Female	119 (39.3)	31 (26.1)	88 (73.9)	21 (17.6)		
						
**Age Classes**					<0.001	<0.001
Under 55	90 (29.7)	10 (11.1)	80 (88.9)	13 (14.4)		
55–65	72 (23.8)	10 (13.9)	62 (86.1)	19 (26.4)		
65–80	74 (24.4)	36 (48.6)	38 (51.4)	34 (45.9)		
Over 80	67 (22.1)	29 (43.3)	38 (56.7)	8 (11.9)		

**Table 3:** **Blood parameters**. Data are reported as absolute frequency (percentage frequency).

Hematochemical Test		Survived	Deceased	Not Admitted to ICU	Admitted to ICU
***Ionized calcium max***	<4.6 mg/dL	170 (90.4)	66 (82.5)	185 (94.9)	51 (69.9)
	4.6–5.3 mg/dL	17 (9.0)	13 (16.2)	9 (4.6)	21 (28.8)
	>5.3 mg/dL	1 (0.5)	1 (1.2)	1 (0.5)	1 (1.4)
		188	80	195	73
***CRP mean***	≤2.9 mg/L	18 (8.3)	0 (0.0)	17 (7.5)	1 (1.4)
	>2.9 mg/L	199 (91.7)	84 (100.0)	211 (92.5)	72 (98.6)
		217	84	228	73
***CRP min***	≤2.9 mg/L	127 (58.5)	3 (3.6)	113 (49.6)	17 (23.3)
	>2.9 mg/L	90 (41.5)	81 (96.4)	115 (50.4)	56 (76.7)
		217	84	228	73
***Total bilirubin min***	<0.20 mg/dL	4 (1.9)	0 (0.0)	4 (1.8)	0 (0.0)
	0.20–1.00 mg/dL	206 (97.2)	76 (90.5)	213 (95.5)	69 (94.5)
	>1.00 mg/dL	2 (0.9)	8 (9.5)	6 (2.7)	4 (5.5)
		212	84	223	73
***Erythrocytes max***	<4.54 $\times 10^{6}/\mu L$ (M) <3.85 $\times 10^{6}/\mu L$ (F)	52 (23.9)	38 (45.2)	60 (26.2)	30 (41.1)
	4.54–5.78 $\times 10^{6}/\mu L$ (M) 3.85–5.16 $\times 10^{6}/\mu L$ (F)	155 (71.1)	39 (46.4)	154 (67.2)	40 (54.8)
	>5.78 $\times 10^{6}/\mu L$ (M) >5.16 $\times 10^{6}/\mu L$ (F)	11 (5.0)	7 (8.3)	15 (6.6)	3 (4.1)
		218	84	229	73
***AST min***	<15 U/L	37 (17.1)	7 (8.3)	31 (13.7)	13 (17.8)
	15–37 U/L	160 (74.1)	47 (56.0)	164 (72.2)	43 (58.9)
	>37 U/L	19 (8.8)	30 (35.7)	32 (14.1)	17 (23.3)
		216	84	227	73

**Table 4:** **Feature selection results for death and admission to ICU**. The table displays the statistical information of the different features filtered according to the logit coefficient shown in the last column, and the *p*-value for both outcomes.

Hematochemical Test		Mean ± Std	Median ± IQR	Min–Max	N	*p*-Value U Test	Logit Coeff
***Ionized calcium max***	Overall	4.2 ± 0.4	4.1 ± 0.3	3.2–7.7	268		
	Survived	4.2 ± 0.3	4.1 ± 0.3	3.2–5.4	188	0.304	−3.178
	Deceased	4.2 ± 0.5	4.2 ± 0.5	3.5–7.7	80		
	Not admitted to ICU	4.1 ± 0.3	4.1 ± 0.2	3.2–5.4	195	0.003	5.629
	Admitted to ICU	4.4 ± 0.5	4.3 ± 0.4	3.6–7.7	73		
***CRP mean***	Overall	66.9 ± 69.7	42.5 ± 76.4	2.9–332.0	301		
	Survived	36.8 ± 32.9	30.2 ± 38.8	2.9–169.4	217	<0.001	4.670
	Deceased	144.7 ± 79.0	137.0 ± 94.9	3.9–332.0	84		
	Not admitted to ICU	47.3 ± 53.0	31.4 ± 49.8	2.9–332.0	228	<0.001	4.169
	Admitted to ICU	128.1 ± 79.9	119.5 ± 92.3	2.9–330.2	73		
***CRP min***	Overall	29.1 ± 52.5	4.6 ± 19.9	2.9–301.0	301		
	Survived	8.0 ± 15.2	2.9 ± 3.9	2.9–142.0	217	<0.001	3.252
	Deceased	83.4 ± 72.2	63.8 ± 119.2	2.9–301.0	84		
	Not admitted to ICU	19.4 ± 41.2	3.1 ± 7.8	2.9–301.0	228	<0.001	7.854
	Admitted to ICU	59.2 ± 70.2	19.8 ± 93.5	2.9–295.0	73		
***Total bilirubin min***	Overall	0.47 ± 0.40	0.40 ± 0.20	0.10–5.90	296		
	Survived	0.41 ± 0.20	0.40 ± 0.20	0.10–1.60	212	<0.001	2.999
	Deceased	0.62 ± 0.66	0.50 ± 0.30	0.20–5.90	84		
	Not admitted to ICU	0.43 ± 0.24	0.40 ± 0.20	0.10–1.60	223	0.009	4.104
	Admitted to ICU	0.58 ± 0.69	0.40 ± 0.20	0.20–5.90	73		
***Erythrocytes max***	Overall	4.5 ± 0.6	4.6 ± 0.8	2.6–6.8	302		
	Survived	4.6 ± 0.5	4.6 ± 0.6	3.1–6.6	218	0.005	2.908
	Deceased	4.4 ± 0.8	4.3 ± 0.9	2.6–6.8	84		
	Not admitted to ICU	4.6 ± 0.6	4.6 ± 0.7	2.6–6.8	229	0.588	4.105
	Admitted to ICU	4.5 ± 0.6	4.5 ± 0.8	3.3–6.2	73		
***AST min***	Overall	26.8 ± 15.0	23.0 ± 15.0	7.0–115.0	300		
	Survived	23.5 ± 10.5	21.0 ± 11.3	7.0–74.0	216	<0.001	3.313
	Deceased	35.3 ± 20.7	31.0 ± 22.3	8.0–115.0	84		
	Not admitted to ICU	25.9 ± 14.0	22.0 ± 14.0	9.0–115.0	227	0.279	7.477
	Admitted to ICU	29.4 ± 17.6	24.0 ± 20.0	7.0–89.0	73		

**Table 5:** **Risk factors for both outcomes: Cox regression analysis**. For each feature, the first row refers to the mortality risk, whereas the second row refers to the admission to ICU.

Hematochemical Test	Normality Range	log(HR)	95% CI log(HR)	HR	95% CI HR	*p*
***CRP mean***	<2.9 mg/L	Not significant				
		1.061	[−0.957, 3.080]	2.890	[0.384, 21.757]	0.303
***CRP min***	<2.9 mg/L	2.888	[1.879, 3.897]	17.963	[6.548, 49.277]	<0.001
		0.582	[0.000, 1.163]	1.789	[1.000, 3.200]	0.050
***Erythrocytes max***	4.54–5.78 $\times 10^{6}/\mu L$ (M) 3.85–5.16 $\times 10^{6}/\mu L$ (F)	0.568	[0.132, 1.004]	1.765	[1.141, 2.729]	0.011
		0.393	[−0.111, 0.897]	1.481	[0.895, 2.452]	0.127
***Total bilirubin min***	0.20–1.00 mg/dL	0.435	[−0.317, 1.188]	1.545	[0.728, 3.279]	0.257
		0.321	[−0.712, 1.355]	1.379	[0.491, 3.876]	0.542
***AST min***	15–37 U/L	0.281	[−0.161, 0.722]	1.324	[0.851, 2.059]	0.213
		0.192	[−0.290, 0.674]	1.211	[0.748, 1.962]	0.436
***Ionized calcium max***	4.6–5.3 mg/dL	0.098	[−0.497, 0.692]	1.103	[0.609, 1.998]	0.747
		−1.293	[−1.843, −0.744]	0.274	[0.158, 0.475]	<0.001

## Data Availability

The data presented in this study are available upon request from the corresponding author.

## Supplementary Materials

The following are available online at https://www.mdpi.com/article/10.3390/s21248503/s1.

## Abbreviations

The following abbreviations are used in this manuscript:

ARDS	acute respiratory distress syndrome
AST	aspartate aminotransferase
AUC	area under the curve
CI	confidence interval
COVID-19	Coronavirus disease 2019
CRP	C-reactive protein
DL	deep learning
DT	decision tree
FN	false negative
FP	false positive
GNB	Gaussian naive Bayes
GRU	gated recurrent unit
HR	hazard ratio
ICU	intensive care unit
IQR	interquartile range
ISTS	irregularly sampled time series
KNN	K-nearest neighbors
LSTM	long short-term memory
ML	machine learning
PCA	principal component analysis
RBC	red blood cells
RF	random Forest
RNN	recurrent neural network
ROC	receiver operating characteristic
RT-PCR	reverse transcription-polymerase chain reaction
SARS-CoV-2	Severe acute respiratory syndrome coronavirus 2
SVM	Support vector machine
T-LSTM	time aware long-short term memory
t-SNE	t-distributed stochastic neighbor embedding
TN	true negative
TP	true positive

## Appendix A. Implementation Details

In regard to the architecture of the ML classifiers, the exhaustive grid search technique has been implemented on a defined subset of the hyperparameters, each in a specific range of possible values, in order to limit the searching time of the k-fold cross-validation procedure (k = 10). The aim of this phase was to optimize the accuracy, resulting in a subset of optimal hyperparameters for each classifier. Then, the classifiers were validated on the hold-out test set, as described in Section 5.3. The setting of hyperparameters has been performed twice, for the death outcome and the admission to ICU outcome.

The hyperparameters tuned for the DT were: the maximum depth of the tree (max_depth), which has been optimized in a range from 1 to 8; the criteria used to measure the quality of a split (criterion), which could be either gini or entropy; the strategy used to choose the split at each node (splitter), which could be random or best.

The only hyperparameter tuned for the GNB classifier was the portion of the largest variance of all features that are added to variance for calculation of the stability (var_smoothing), which was optimized in a range between $10^{-10}$ and 10, with 10 steps.

The hyperparameters tuned for the SVM were: the regularization parameter (C), chosen from the set {1,10,100,1000}; the kernel type used in the algorithm (kernel), chosen from the set {linear,poly,rbf,sigmoid}; the kernel coefficient (gamma), which could be either $10^{-3}$ or $10^{-4}$ (this parameter is set only when the kernel is not linear).

The hyperparameters tuned for the KNN were: the number of neighbors to use (n_neighbors), in range from 1 to 10; the distance metric used by the tree (metric), tuned from the set {euclidean,manhattan,chebyshev}.

The hyperparameters tuned for the RF were: the criterion, as for the DT; the max_depth, tuned in the range from 1 to 10; the bootstrap dichotomous value, to decide if exploiting all the sample test data, or only the bootstrap sample.

The hyperparameters tuned for the AdaBoost were: the weights applied to each classifier at each iteration (learning_rate), in a range from $10^{-4}$ to 1; the maximum number of estimators used (n_estimators), in a range from 10 to 100.

From our experiments, the optimal configuration of hyperparameters for each classifier is as listed below.

- Death outcome best configuration:
  – DT—criterion: gini; max_depth: 2; splitter: best.
  – GNB—var_smoothing: 0.001.
  – SVM—C: 1000; kernel: rbf; gamma: 0.001.
  – KNN—metric: euclidean; n_neighbors: 5.
  – RF—bootstrap: true; criterion: gini; max_depth: 7.
  – AdaBoost—learning_rate: 0.01; n_estimators: 70.
- Admission to ICU outcome best configuration:
  – DT—criterion: entropy; max_depth: 2; splitter: best.
  – GNB—var_smoothing: 0.01. SVM – C: 1000; kernel: linear.
  – KNN—metric: euclidean; n_neighbors: 5.
  – RF—bootstrap: true; criterion: gini; max_depth: 7.
  – AdaBoost—learning_rate: 0.1; n_estimators: 80.
